# How often is prophylactic parastomal mesh placement performed after rectal resection without sphincter preservation? An analysis of German nationwide hospital discharge data among 41,697 patients

**DOI:** 10.1007/s10029-023-02887-9

**Published:** 2023-10-16

**Authors:** C. Paasch, E. Kobelt, S. Lünse, S. Heisler, R. Lorenz, R. Hunger, R. Mantke

**Affiliations:** 1 Department of Surgery, Brandenburg Medical School, University Hospital Brandenburg/Havel, 14770 Brandenburg, Germany; 2 Faculty of Health Science Brandenburg, Brandenburg Medical School, University Hospital Brandenburg/Havel, 14770 Brandenburg, Germany; 3 Hernia Center 3+CHIRURGEN, Berlin, Germany; 4 Clinic for General and Visceral Surgery, University Hospital Brandenburg an der Havel, Brandenburg Medical University, Hochstraße 29, 14770 Brandenburg an der Havel, Germany

**Keywords:** Parastomal hernia, Rectum extirpation, Guideline, Non-absorbable mesh, Prophylactic mesh placement

## Abstract

**Purpose:**

The European Hernia Society guidelines of parastomal hernias, published in 2017, strongly recommend prophylactic synthetic non-absorbable mesh upon the construction of a permanent end colostomy to reduce the incidence of parastomal hernias. This study aims to evaluate the implementation of the guidelines in Germany.

**Methods:**

This is a retrospective multicentric analysis conducted in December 2022 at the University Hospital Brandenburg an der Havel. Anonymous data on rectal resection without sphincter preservation in the period 2010–2020 were extracted from the German nationwide hospital discharge data set. Individuals with a hernia and < 18 years old were excluded. Another exclusion criterion was a performed colectomy or proctocolectomy with an ileoanal pouch and placement of an absorbable mesh. The primary endpoint was the annual rate of prophylactic parastomal mesh placement following rectal resection without sphincter preservation in Germany. Cases reporting both non-absorbable mesh placement and rectal resection without sphincter preservation were considered prophylactic mesh insertions.

**Results:**

A total of 41,697 patients received a rectal resection without sphincter preservation and without non-absorbable mesh placement. Among these individuals, 27,089 were male and 14,608 were female. The rate of reoperations (3.1%) and the length of hospital stay (25.3 days ± 19.32) remained almost constant during these 10 years. The rate of prophylactic mesh placement was increasing from 0.2% (*n* = 8) in 2010 to 6.4% (*n* = 198) in 2020.

**Conclusions:**

Currently, only the minority of patients who have undergone rectal resection without sphincter preservation receive prophylactic mesh insertion.

**Supplementary Information:**

The online version contains supplementary material available at 10.1007/s10029-023-02887-9.

## Introduction

A parastomal hernia (PH) frequently occurs following rectum extirpation when conducting an end colostomy. In literature, an incidence of up to 60% has been published [1, 2]. To reduce the incidence of PH, prophylactic mesh placement has been studied in a total of 13 randomized clinical trials over the past 14 years (table S1) [2]. The authors revealed a significant reduction in PH occurrences after prophylactic mesh placement. As a result, the European Hernia Society created a guideline in 2017 based on these findings [3]. The guideline strongly recommends prophylactic synthetic non-absorbable mesh upon construction of an end colostomy.

To achieve a reduced rate of PHs, it is important to truly implement the guidelines. It is necessary to determine if additional promotion is required. Thus, this study aimed to evaluate the implementation of the guidelines in Germany based on German nationwide hospital discharge data.

## Methods

This is a retrospective multicentric analysis conducted in December 2022 at the University hospital Brandenburg an der Havel. Hospitals and patients are anonymized. Neither institutes nor several hospital stays of patients can be identified. Thus, ethical approval and patient consent were not needed for the analysis at hand.

The primary endpoint was the annual rate of prophylactic parastomal mesh placement following rectal resection without sphincter preservation in Germany from 2010 to 2020. Secondary endpoints were the length of hospital stay and the rate of reoperation.

### Primary endpoint definition

The data of individuals who received a rectal resection, without sphincter preservation, and with the conduction of a permanent end colostomy were extracted. Patients with a diagnosis of a hernia as well as individuals under the age of 18 were excluded.

**Fig. 1:**
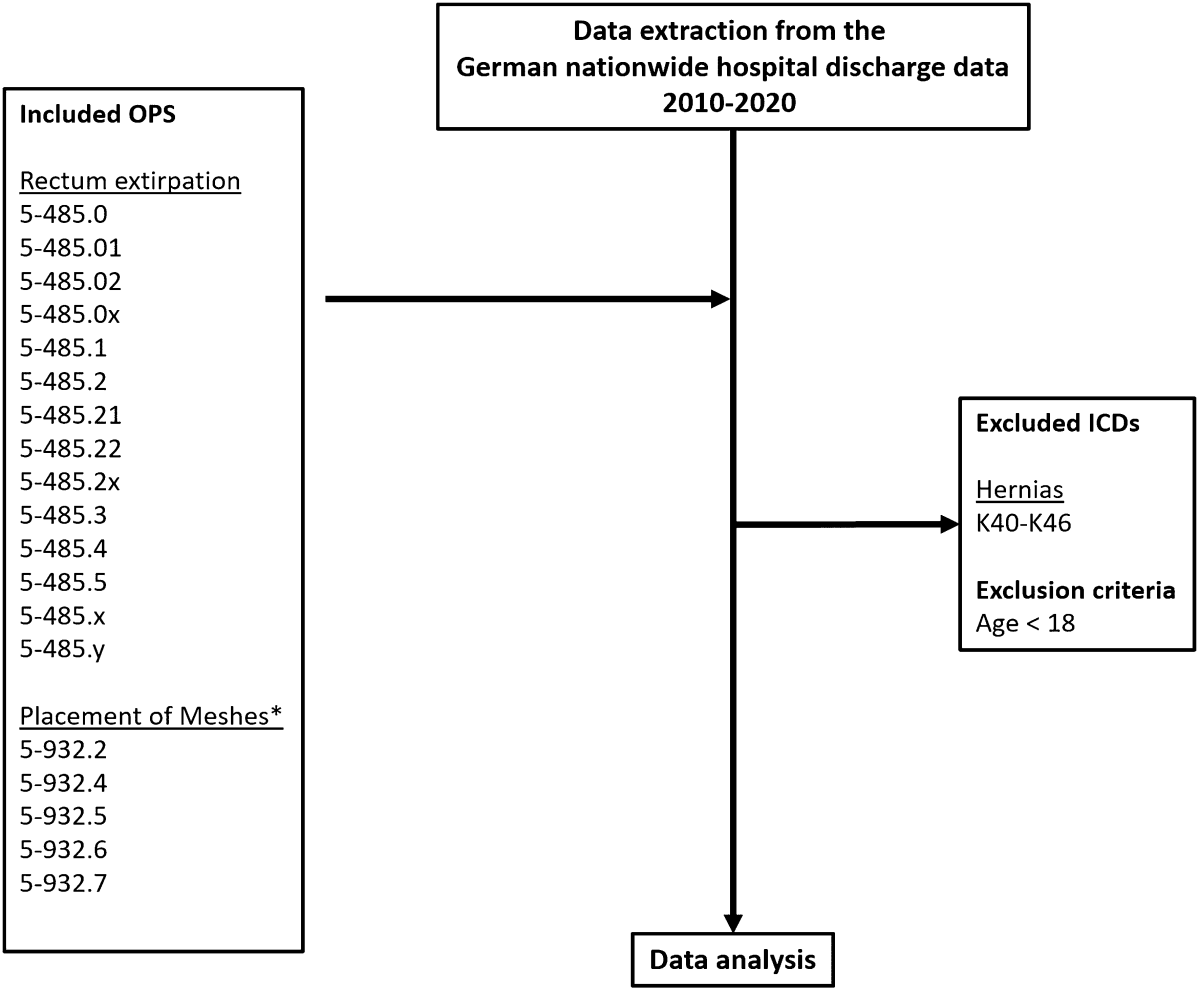
Flowchart of data analysis; ICD International Classification of Diseases 10; OPS Surgical Operation and Procedure classification system

To identify cases in which prophylactic mesh placement simultaneously took place, a search was performed for OPS (Surgical Operation and Procedure code classification system) numbers of meshes among the primary extracted data (Table 1 and Fig. 1). Cases reporting both non-absorbable mesh and rectal resection without sphincter preservation were considered prophylactic mesh insertions.

**Table 1:** Procedures and diagnoses

Definition	Condition	Procedure/diagnosis codes
*Inclusion*		
	Rectal resection without sphincter	5-485.0, 5-485.01, 5-485.02
		5-485.0x, 5-485.1, 5-485.2
		5-485.21, 5-485.22, 5-485.2x
		5-485.3, 5-485.4, 5-485.5
		5-485.x, 5-485-y
	Mesh placement	5-932.2, 5-932.4
		5-932.5, 5-932.6, 5-932.7
*Exclusion*		
	All types of a hernia	K40, K41, K42, K43
		K44, K45, K46
	Colectomy or proctocolectomy with ileoanal pouch	5-456.1, 5-456.10, 5-456.17, 5-456.18, 5-456.1
	Absorbable and biological mesh placement	5-932.8, 5-932.9, 5-932.1

Sub-classifications of respective codes are included

Due to the pelvic placement of absorbable meshes in some cases of rectum extirpation, the OPS numbers for absorbable meshes were excluded. Figure 1 depicts the data flowchart with information on exclusion and inclusion criteria.

### Study design

The DRG dataset includes all stationary inpatient occurrences from all hospitals in Germany that take part in this compensation system. A few hospitals (military hospitals) and cases (psychosomatic, psychiatric) are excluded from the system.

The information on each inpatient episode is compulsorily collected by the individual hospitals and forwarded to the Institute for the Hospital Remuneration System. There, the data records are checked for plausibility, anonymized, and finally forwarded to the German Federal Statistical Office. This office uses controlled remote data processing and makes patients' information available for scientific research.

The obligation to collect data, the criteria to be collected, the individual processing steps, and the possible uses are specified in detail in the law.

At the patient level, the dataset includes demographic baseline information (gender, age), information on primary and secondary diagnoses [according to the German modification of the International Classification of Diseases 10 (ICD-10)], and medical procedures performed. The dataset further contains information on each hospital stay (type of admission, length-of-stay, and discharge status).

### Statistical analysis

Quantitative data were expressed as mean ± standard deviation (SD) and qualitative data as frequency and percentage. The primary endpoint was analyzed using a chi-squared test for trend in proportions (Cochran–Armitage test) with equally spaced score values for observation years. The impact of prophylactic mesh placement on the secondary endpoints of mortality and reoperation rate were assessed with chi-square tests. The length-of-stay was compared between the two study groups with a Mann–Whitney test.

Statistical analyses were conducted using R (version 4.2.3., The R Foundation, Vienna, Austria). All tests were two-sided and *p* values < 0.05 were considered indicative of statistical significance.

## Results

A total of 41,715 cases were extracted from the German nationwide hospital discharge data set, of which 13 patients had to be excluded due to a hernia, three individuals due to their age of under 18 years, and two individuals without information on gender.

**Fig. 2:**
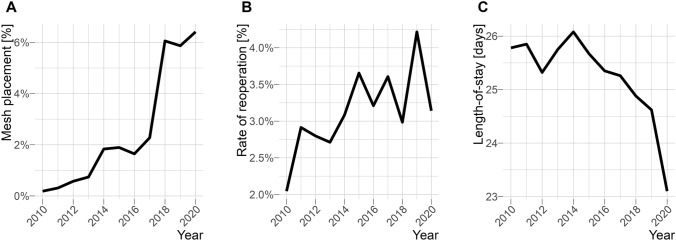
The rate of mesh placement (**A**), reoperation (**B**), and the length of hospital stay is depicted in figure

A total of 41,697 records of rectal resections without sphincter preservation from 1077 hospitals were used for the analysis. Among these individuals, 27,089 were male and 14,608 were female. The age was on average 68.5 years (± 11.5). A total of 34,010 (81.6%) individuals underwent elective surgery whereas 7,025 patients were operated on to an emergency. In 968 cases, a simultaneously non-absorbable mesh placement took place (2.3%). A significant increase in the proportion of mesh placements during the analysis period was registered (χ^2^ (df = 1) = 747.6, *p* < 0.001). The frequency of placement of non-absorbable meshes after rectal resection without sphincter preservation between 2010 and 2020 is summarized in Table 2 and Fig. 2.

**Table 2:** Frequency of placement of non-absorbable meshes after rectal resection without sphincter preservation

Variable	Total	2010	2011	2012	2013	2014	2015	2016	2017	2018	2019	2020
Total	41,697 (100%)	4303 100%	4187 100%	4181 100%	4056 100%	3926 100%	3803 100.0%	3707 100%	3604 100%	3451 100%	3391 100%	3088 100%
5-932.2|3|4|6|7	968 (2.3%)	8 (0.2%)	13 (0.3%)	24 (0.6%)	30 (0.7%)	72 (1.8%)	72 (1.9%)	61 (1.6%)	82 (2.3%)	209 (6.1%)	199 (5.9%)	198 (6.4%)

German Surgical Operation and procedure code for non-absorbable meshes: 5-932.2|3|4|6|7

The mean length of hospital stay was 25.3 days (± 19.3). Patients with prophylactic mesh placement had an average length-of-stay of 29.3 days (± 25.1) and patients without prophylactic mesh placement had 25.1 days (± 18.9). The difference was significant, *t*(df = 41,695) = 9.9, *p* < 0.001.

The reoperation rate was 3.1% (*n* = 1289). Stratified by group, the reoperation rate was slightly higher in the prophylactic mesh group (3.8% vs. 3.1%); however, the groups did not differ significantly (χ^2^ (df = 1) = 1.77, *p* = 0.18). The overall in-hospital mortality rate was 4.2% (*n* = 1749), with no substantial differences between patients with and without mesh placement [3.8% and 4.2%, respectively; χ^2^ (df = 1) = 0.34, *p* = 0.56].

## Discussion

Clinical guidelines are systematically developed statements to support decision-making by physicians, others involved in the health care system, and patients. The goal is to provide appropriate health-related care in specific clinical situations. They have a long tradition. For example, the guideline on brain and spinal cord injuries was written already in 1945 and it is the first PubMed-listed guideline. Since then, more than 70,000 scientific articles on guidelines have been published. As is well known, these systematically developed statements are usually based on clinical trials and publications with high scientific evidence. In other words, a group of experts in a particular medical field evaluate the literature and summarize their findings for readers. Although the benefits of following or at least recognizing guidelines are crystal clear, their implementation usually requires many years and publicity [4, 5]. As an example, guidelines from 2002 on preoperative tests before cataract surgery are not recommended. However, Chen et al. (2015), in an observational study of 440,857 patients, found that these tests were still part of the daily routine in 54% of cases [4]. The authors revealed these findings 13 years after the guideline publication.

Appropriately, only 6.4% of patients currently receive prophylactic mesh after rectal resection without sphincter preservation. The clinical studies on this topic and the European Hernia Society guideline published in 2017 resulted in an increase from 0.2% to 6.4% for prophylactic mesh insertion (Fig. 2). These findings are consistent with a 2019 survey on this topic of 141 colorectal surgeons, two-thirds of whom perform prophylactic mesh reinforcement of the permanent stoma in less than 5%. Only 10% of surgeons surveyed used prophylactic mesh in nearly 75–100% of their patients [6].

The data we collected help to evaluate the implementation process of this guideline. Our findings suggest an insufficient implementation of this recommendation. The reasons for this are most likely complex. A possible lack of guideline publicity and promotion has to be mentioned. Moreover, the actual German S3-guideline on colorectal carcinoma from 2015 (currently under review) does not recommend prophylactic mesh insertion [7]. The guideline was published prior to the guidelines on PH. Furthermore, its insufficient implementation might be the operation time of rectal resection without sphincter preservation. Usually lasting up to 4 h, it is often a time-consuming and strenuous surgical procedure [8]. The potentially time-consuming mesh insertion at the end of surgery could be an obstacle to the implementation of the guideline recommendation for prophylactic mesh insertion. The *Keyhole* and* Sugarbaker* techniques are often conducted for this mesh placement. Both approaches may last up to 2 h [9]. A further reason might be also the fear of mesh infection. This seems not comprehensible, as a 2018 Cochrane analysis failed to identify a mesh infection rate among participants (*n* = 844) in ten randomized clinical trials [10]. In the present analysis, the increasing rate of mesh placement did not lead to an increased rate of reoperation, mortality, or hospital stay. Mesh infection would most likely lead to an increased rate of reoperation. Thus, it can be assumed that prophylactic mesh placement per the Cochrane analysis by Jones et al. in 2018 might be safe [10].

Another reason for not using prophylactic mesh insertion could be the current literature questioning the benefit of prophylactic mesh insertion. In this regard, an updated meta-analysis from 2021 (11 × randomized clinical trials, *n* = 1097) focused on studies with low risk of bias, among others namely the PREVENT and STOMAMESH trial [11–14].

According to this, the benefit of prophylactic mesh insertion (in the retrorectal space) was lost in terms of reducing to the rate of parastomal hernias. However, the rate of parastomal hernias was for example in the PREVENT and STOMAMESH trial without significance higher (PREVENT trial: non-mesh group, *n* = 20, 27.8% versus mesh group, *n* = 29, 37.2%) [11].

Furthermore, in a retrospective analysis (*n* = 116), Tackström et al. (2022) showed that a prophylactic stoma mesh was a risk factor for the development of rectus abdominis muscle atrophy. On the other hand, these patients were not at risk for parastomal hernia compared to the non-mesh group [15]. The inadequate implementation and adherence that we have identified may lead to the need to further promote the guidelines. Next to the announcement of its publication and (free) accessibility to national and international conferences, further tools should be used. Some authors reported that social media and YouTube could also be helpful, as these sources address the "lack of time" barrier to access guidelines [16]. Loeb et al. (2017) evaluated the use of Twitter to disseminate and evaluate adherence to clinical guidelines by the European Association of Urology. The authors found that 8709 posts reached a total of 9,117,746 views within approximately 18 months. Surveys (*n* = 16 within 6 months) were also conducted. They resulted in an increase in guideline adherence [17]. On the other hand, guidelines, announcements, and publications are frequently published by the European Hernia Society, e.g., via Twitter and its homepage. Perhaps, in the future, only the most important information should be disclosed via social media to avoid being overwhelmed by too much information.

Compared to other European countries, the length of hospital stay (25.3 days, ± 19.3) seems to be higher. One explanation for this could be the lower rate of minimally invasive (assisted) surgery in Germany. Unfortunately, our data do not provide any information about this.

As a limitation of the study, reporting of both rectal resections without sphincter preservation and placement of an absorbable mesh was not considered prophylactic mesh placement. As noted in the Methods section, OPS numbers were excluded based on pelvic absorbable mesh placement. Nevertheless, it is possible that some surgeons used an absorbable mesh for prophylactic placement contrary to guideline recommendations. To that, our analysis revealed that absorbable meshes were more frequently inserted after guideline publication [1.1% (*n* = 47) in 2010 to 3.9% (*n* = 119 = in 2020; Fig. S1)]. Since the publication aimed to analyze the implementation of guidelines—the placement of non-absorbable mesh is clearly recommended—it is reasonable to exclude the OPS code for non-absorbable in terms of primary endpoint determination. Another limitation could be the fact that, in some cases, surgeons forgot to report the placement of the mesh. On the other hand, this is unlikely, especially regarding reimbursement.

## Conclusion

Currently, only 6.4% of patients who have undergone rectal resection without sphincter preservation received prophylactic mesh insertion. An update of the guideline by the European Hernia Society could be useful due to the long-term results published on this topic in recent years.

### Supplementary Information

Below is the link to the electronic supplementary material.

Figure S1 Placement of non-absorbable and absorbable meshes when conducting rectal resection without sphincter preservation from 2010 to 2020. Supplementary file1 (PNG 89 KB)

Supplementary file2 (DOCX 36 KB)

## Data Availability

The data are available based on reasonable request.

## References

[1] Carne PW, Robertson GM, Frizelle FA (2003). Parastomal hernia. Br J Surg.

[2] McKechnie T, Lee J, Lee Y, Doumouras A, Amin N, Hong D, Eskicioglu C (2022). Prophylactic mesh for prevention of parastomal hernia following end colostomy: an updated systematic review and meta-analysis of randomized controlled trials. J Gastrointest Surg.

[3] Antoniou SA, Agresta F, Garcia Alamino JM, Berger D, Berrevoet F, Brandsma HT, Bury K, Conze J, Cuccurullo D, Dietz UA, Fortelny RH, Frei-Lanter C, Hansson B, Helgstrand F, Hotouras A, Janes A, Kroese LF, Lambrecht JR, Kyle-Leinhase I, Lopez-Cano M, Maggiori L, Mandala V, Miserez M, Montgomery A, Morales-Conde S, Prudhomme M, Rautio T, Smart N, Smietanski M, Szczepkowski M, Stabilini C, Muysoms FE (2018). European Hernia Society guidelines on prevention and treatment of parastomal hernias. Hernia.

[4] Chen CL, Lin GA, Bardach NS, Clay TH, Boscardin WJ, Gelb AW, Maze M, Gropper MA, Dudley RA (2015). Preoperative medical testing in medicare patients undergoing cataract surgery. N Engl J Med.

[5] Partridge MR (2003). Translating research into practice: how are guidelines implemented?. Eur Respir J Suppl.

[6] Aslam MI, Rubio-Perez I, Smart NJ, Singh B (2019). A survey on practices for parastomal hernia prevention and repair among ESCP surgeons. Hernia.

[7] Deutsche Gesellschaft für Gastroenterologie V-uS, (DGVS) (2019) S3-Leitlinie Kolorektales Karzinom. AWMF-Registernummer: 021/007OL 10.1055/s-0033-1350264 23955142

[8] Han JG, Wang ZJ, Wei GH, Gao ZG, Yang Y, Zhao BC (2012). Randomized clinical trial of conventional versus cylindrical abdominoperineal resection for locally advanced lower rectal cancer. Am J Surg.

[9] Makarainen-Uhlback E, Vironen J, Vaarala M, Nordstrom P, Valikoski A, Kossi J, Falenius V, Kechagias A, Mattila A, Ohtonen P, Scheinin T, Rautio T (2021). Keyhole versus Sugarbaker techniques in parastomal hernia repair following ileal conduit urinary diversion: a retrospective nationwide cohort study. BMC Surg.

[10] Jones HG, Rees M, Aboumarzouk OM, Brown J, Cragg J, Billings P, Carter B, Chandran P (2018). Prosthetic mesh placement for the prevention of parastomal herniation. Cochrane Database Syst Rev.

[11] Brandsma HT, Hansson BM, Aufenacker TJ, de Jong N, Engelenburg KCV, Mahabier C, Donders R, Steenvoorde P, de Vries Reilingh TS, Leendert van Westreenen H, Wiezer MJ, de Wilt JHW, Rovers M, Rosman C (2023). Prophylactic mesh placement during formation of an end-colostomy: long-term randomized controlled trial on effectiveness and safety. Ann Surg.

[12] Odensten C, Strigard K, Rutegard J, Dahlberg M, Stahle U, Gunnarsson U, Nasvall P (2019). Use of prophylactic mesh when creating a colostomy does not prevent parastomal hernia: a randomized controlled trial-STOMAMESH. Ann Surg.

[13] Sahebally SM, Lim TZ, Azmir AA, Lu CT, Doudle M, Naik A, Nolan G, Papen MV (2021). Prophylactic mesh placement at index permanent end colostomy creation to prevent parastomal hernia-an updated meta-analysis. Int J Colorectal Dis.

[14] Mohiuddin S, Hollingworth W, Rajaretnam N, Reeves BC, Smart NJ (2021). Use of prophylactic mesh during initial stoma creation to prevent parastomal herniation: a systematic review and meta-analysis of randomised controlled trials. Colorectal Dis.

[15] Täckström S, Chabok A, Smedh K, Nikberg M (2022). Use of prophylactic stoma mesh is a risk factor for developing rectus abdominis muscle atrophy. Hernia.

[16] Rayner S, Marlow G, Leslie SJ (2017). YouTube: a solution to increased dissemination of guidelines?. Med Educ.

[17] Loeb S, Roupret M, Van Oort I, N'Dow J, van Gurp M, Bloemberg J, Darraugh J, Ribal MJ (2017). Novel use of Twitter to disseminate and evaluate adherence to clinical guidelines by the European Association of Urology. BJU Int.

