# Supplementary figures and images for: How often is prophylactic parastomal mesh placement performed after rectal resection without sphincter preservation? An analysis of German nationwide hospital discharge data among 41,697 patients
Source: Hernia. 2023 Oct 16;28(1):9–15. doi: 10.1007/s10029-023-02887-9 (PMC10891180; doi:10.1007/s10029-023-02887-9)

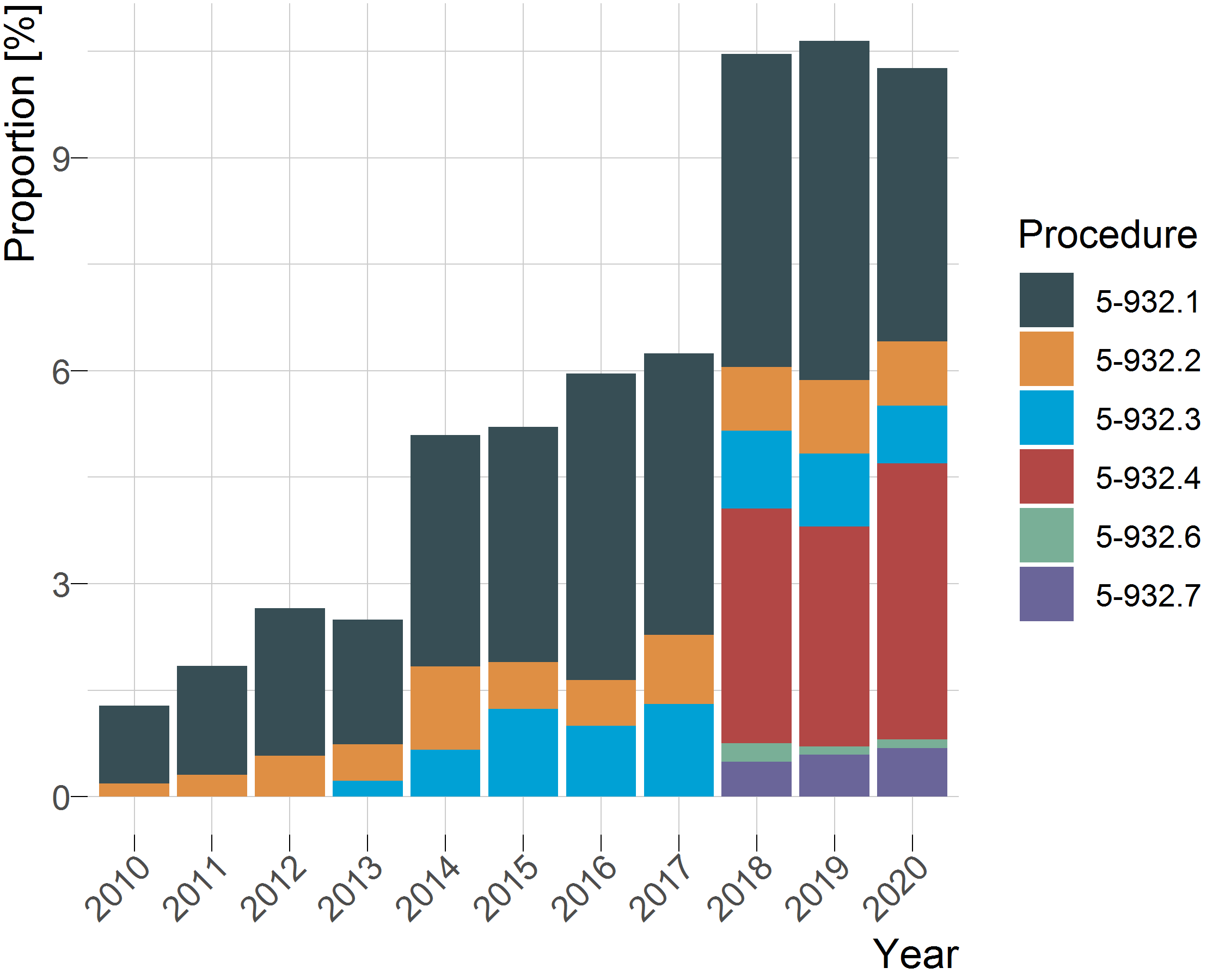

Supplement: Supplementary file 1 — Placement of non-absorbable and absorbable meshes when conducting rectal resection without sphincter preservation from 2010 to 2020. Supplementary file1 (PNG 89 KB) [file 10029_2023_2887_MOESM1_ESM.png]
