# Supplementary material for: How often is prophylactic parastomal mesh placement performed after rectal resection without sphincter preservation? An analysis of German nationwide hospital discharge data among 41,697 patients
Source: Hernia. 2023 Oct 16;28(1):9–15. doi: 10.1007/s10029-023-02887-9 (PMC10891180; doi:10.1007/s10029-023-02887-9)
Supplement: Supplementary file 2 — Supplementary file2 (DOCX 36 KB) [file 10029_2023_2887_MOESM2_ESM.docx]

**Supplementary Table S1** Overview of randomized controlled trials (RCTs) comparing the use of a prophylactic mesh versus no prophylactic mesh for end colostomy after colorectal resection (N number of patients, SD standard deviation, CRC colorectal cancer).

| Year | Study | *N* | Arm (n) | Mean age, years (SD) | Index operation | Indication for index operation | Parastomal hernia rate | Follow up |
| --- | --- | --- | --- | --- | --- | --- | --- | --- |
| **2009** | Jänes et al. [1] | 54 | Mesh (27)  No Mesh (27) | 70  71 | -  - | CRC, diverticulitis, IBD  CRC, diverticulitis, IBD, other | **13.3%** (2/15)  **81%** (17/21) | 5 years |
|  | Serra-Aracil et al. [2] | 54 | Mesh (27)  No Mesh (27) | 67.5 (8.8)  67.2 (9.7) | APR  APR | CRC CRC | **22.2%** (6/27)  **44.4%** (14/27) | 29 months |
| **2012** | Lopez-Cano et al. [3] | 36 | Mesh (19)  No Mesh (17) | 72.2 (7.6)  65.9 (13.9) | APR  APR | CRC  CRC | **50%** (9/18)  **93.8%** (15/16) | 12 months |
| **2014** | Tarcoveanu et al. [4] | 42 | Mesh (20)  No Mesh (22) | -  - | APR  APR | CRC  CRC | **0%** (0/20)  **27.2%** (6/22) | 20 months |
|  | Fleshman et al. [5] | 113 | Mesh (55)  No Mesh (58) | 60.2 (13.6)  59.1 (14.4) | APR, proctocolectomy, colostomy, ileostomy, LAR, sigmoid resection, stoma relocation  APR, proctocolectomy, colostomy, ileostomy, LAR, sigmoid resection, stoma relocation | CRC, Crohn’s, UC, fecal incontinence, anal cancer, constipation, IBS, rectal prolapse  CRC, Crohn’s, UC, fecal incontinence, anal cancer, constipation, IBS, congenital GI abnormality, radiation proctitis | **10.2%** (5/49)  **13.2%** (7/53) | 24 months |
| **2015** | Lambrecht et al. [6] | 58 | Mesh (32)  No Mesh (26) | 64 (4.0)  63 (4.1) | APR, pelvic exenteration, Hartmann’s  APR | CRC  CRC | **6%** (2/32)  **46%** (12/26) | 40 months |

**Supplementary Table S1** (continued)

| Year | Study | *N* | Arm (n) | Mean age, years (SD) | Index operation | Indication for index operation | Parastomal hernia rate | Follow up |
| --- | --- | --- | --- | --- | --- | --- | --- | --- |
| **2015** | Vierimaa et al. [7]  Brandsma et al. [8] | 70  133 | Mesh (35)  No Mesh (35)  Mesh (67)  No Mesh (66) | 67.1 (10.7)  65.1 (11.7)  64.1  63.6 | APR  APR  APR  APR | CRC  CRC  CRC, fecal incontinence, IBD, other  CRC, fecal incontinence, IBD, other | **51.4%** (18/34)  **53.1%** (17/32)  **0.7%** (1/141)  **2.7%** (4/146) | 12 months  3 months |
| **2016** | Lopez-Cano et al. [9] | 52 | Mesh (24)  No Mesh (28) | 70.5 (9.5)  67.3 (13.6) | APR  APR | CRC  CRC | **25%** (6/24)  **64.3%** (18/28) | 26 months |
| **2019** | Odensten et al. [10] | 232 | Mesh (114)  No Mesh (118) | 69.7  69.9 | -  - | CRC, benign disease  CRC, benign disease | **24%** (25/104)  **26%** (28/107) | 12 months |
| **2021** | Prudhomme et al. [11] | 199 | Mesh (98)  No Mesh (101) | 67.2 (12.4)  70.5 (11.1) | -  - | CRC, fecal incontinence, IBD, pelvic reoperation  CRC, fecal incontinence, pelvic reoperation | **31%** (30/70)  **28%** (28/65) | 24 months |
|  | Correa-Martinez et al. [12] | 209 | Mesh (63)  No Mesh (146) | 65.0  63.6 | Colostomy only, Hartmann’s, APR  Colostomy only, Hartmann’s, APR, NR | CRC, benign disease  CRC, benign disease, NR | **39.6%** (23/58)  **44.1%** (56/127) | 12 months |
| **2023** | Ringblom et al. [13] | 232 | Mesh (114)  No Mesh (118) | 69.7  69.8 | APR  APR | CRC  CRC | **42.4%** (42/99)  **44.6%** (45/101) | 3 years |

**References**

1. Janes A, Cengiz Y, Israelsson LA. Preventing parastomal hernia with a prosthetic mesh: a 5-year follow-up of a randomized study. World J Surg. 2009;33(1):118-21; discussion 22-3. doi: 10.1007/s00268-008-9785-4.

2. Serra-Aracil X, Bombardo-Junca J, Moreno-Matias J, Darnell A, Mora-Lopez L, Alcantara-Moral M, et al. Randomized, controlled, prospective trial of the use of a mesh to prevent parastomal hernia. Annals of surgery. 2009;249(4):583-7. doi: 10.1097/SLA.0b013e31819ec809.

3. Lopez-Cano M, Lozoya-Trujillo R, Quiroga S, Sanchez JL, Vallribera F, Marti M, et al. Use of a prosthetic mesh to prevent parastomal hernia during laparoscopic abdominoperineal resection: a randomized controlled trial. Hernia. 2012;16(6):661-7. doi: 10.1007/s10029-012-0952-z.

4. Tarcoveanu E, Vasilescu A, Cotea E, Vlad N, Palaghia M, Danila N, et al. Parastomal hernias -- clinical study of therapeutic strategies. Chirurgia (Bucur). 2014;109(2):179-84.

5. Fleshman JW, Beck DE, Hyman N, Wexner SD, Bauer J, George V, et al. A prospective, multicenter, randomized, controlled study of non-cross-linked porcine acellular dermal matrix fascial sublay for parastomal reinforcement in patients undergoing surgery for permanent abdominal wall ostomies. Diseases of the colon and rectum. 2014;57(5):623-31. doi: 10.1097/DCR.0000000000000106.

6. Lambrecht JR, Larsen SG, Reiertsen O, Vaktskjold A, Julsrud L, Flatmark K. Prophylactic mesh at end-colostomy construction reduces parastomal hernia rate: a randomized trial. Colorectal Dis. 2015;17(10):O191-7. doi: 10.1111/codi.13065.

7. Vierimaa M, Klintrup K, Biancari F, Victorzon M, Carpelan-Holmstrom M, Kossi J, et al. Prospective, Randomized Study on the Use of a Prosthetic Mesh for Prevention of Parastomal Hernia of Permanent Colostomy. Diseases of the colon and rectum. 2015;58(10):943-9. doi: 10.1097/DCR.0000000000000443.

8. Brandsma HT, Hansson BM, Aufenacker TJ, van Geldere D, van Lammeren FM, Mahabier C, et al. Prophylactic mesh placement to prevent parastomal hernia, early results of a prospective multicentre randomized trial. Hernia. 2016;20(4):535-41. doi: 10.1007/s10029-015-1427-9.

9. Lopez-Cano M, Serra-Aracil X, Mora L, Sanchez-Garcia JL, Jimenez-Gomez LM, Marti M, et al. Preventing Parastomal Hernia Using a Modified Sugarbaker Technique With Composite Mesh During Laparoscopic Abdominoperineal Resection: A Randomized Controlled Trial. Annals of surgery. 2016;264(6):923-8. doi: 10.1097/SLA.0000000000001684.

10. Odensten C, Strigard K, Rutegard J, Dahlberg M, Stahle U, Gunnarsson U, et al. Use of Prophylactic Mesh When Creating a Colostomy Does Not Prevent Parastomal Hernia: A Randomized Controlled Trial-STOMAMESH. Annals of surgery. 2019;269(3):427-31. doi: 10.1097/SLA.0000000000002542.

11. Prudhomme M, Rullier E, Lakkis Z, Cotte E, Panis Y, Meunier B, et al. End Colostomy With or Without Mesh to Prevent a Parastomal Hernia (GRECCAR 7): A Prospective, Randomized, Double Blinded, Multicentre Trial. Annals of surgery. 2021;274(6):928-34. doi: 10.1097/SLA.0000000000004371.

12. Correa Marinez A, Bock D, Erestam S, Engstrom A, Kalebo P, Nielsen YW, et al. Methods of Colostomy Construction: No Effect on Parastomal Hernia Rate: Results from Stoma-const-A Randomized Controlled Trial. Annals of surgery. 2021;273(4):640-7. doi: 10.1097/SLA.0000000000003843.

13. Ringblom C, Odensten C, Strigard K, Gunnarsson U, Nasvall P. No Reduction in Parastomal Hernia Rate 3 Years After Stoma Construction With Prophylactic Mesh: Three-year Follow-up Results From STOMAMESH-A Multicenter Double-blind Randomized Controlled Trial. Annals of surgery. 2023;277(1):38-42. doi: 10.1097/SLA.0000000000005537.
